# Supplementary material for: Modeling glioblastoma heterogeneity as a dynamic network of cell states
Source: Mol Syst Biol. 2021 Sep 16;17(9):e10105. doi: 10.15252/msb.202010105 (PMC8444284; doi:10.15252/msb.202010105)
Supplement: Supplementary file 5 — Source Data for Figure 3 [file MSB-17-e10105-s001.zip › Figure3A_sourcedata/GSEA_3065/hallmarks_state6.GseaPreranked.1623416308702/HALLMARK_COMPLEMENT.html]

Details for gene set HALLMARK\_COMPLEMENT[GSEA]

|  || Dataset | state6 |
| Phenotype | NoPhenotypeAvailable |
| Upregulated in class | na\_neg |
| GeneSet | HALLMARK\_COMPLEMENT |
| Enrichment Score (ES) | -0.4286269 |
| Normalized Enrichment Score (NES) | -1.6876631 |
| Nominal p-value | 0.0 |
| FDR q-value | 0.011425123 |
| FWER p-Value | 0.079 |
Table: GSEA Results Summary

  

Fig 1: Enrichment plot: HALLMARK\_COMPLEMENT      
 Profile of the Running ES Score & Positions of GeneSet Members on the Rank Ordered List

  

| PROBE | GENE SYMBOL | GENE\_TITLE | RANK IN GENE LIST | RANK METRIC SCORE | RUNNING ES | CORE ENRICHMENT || 1 | ATOX1 |  |  | 179 | 0.183 | 0.0058 | No |
| 2 | CALM3 |  |  | 435 | 0.130 | -0.0047 | No |
| 3 | MT3 |  |  | 662 | 0.109 | -0.0149 | No |
| 4 | PCLO |  |  | 760 | 0.101 | -0.0115 | No |
| 5 | FYN |  |  | 817 | 0.097 | -0.0039 | No |
| 6 | PPP2CB |  |  | 906 | 0.091 | -0.0010 | No |
| 7 | FDX1 |  |  | 916 | 0.090 | 0.0110 | No |
| 8 | GNG2 |  |  | 930 | 0.090 | 0.0224 | No |
| 9 | CEBPB |  |  | 943 | 0.089 | 0.0339 | No |
| 10 | SIRT6 |  |  | 1222 | 0.075 | 0.0127 | No |
| 11 | IRF2 |  |  | 1642 | 0.059 | -0.0270 | No |
| 12 | USP8 |  |  | 1682 | 0.057 | -0.0231 | No |
| 13 | RHOG |  |  | 1688 | 0.057 | -0.0155 | No |
| 14 | STX4 |  |  | 1765 | 0.055 | -0.0163 | No |
| 15 | RAF1 |  |  | 1924 | 0.050 | -0.0273 | No |
| 16 | ANXA5 |  |  | 1978 | 0.048 | -0.0264 | No |
| 17 | IRF1 |  |  | 2377 | 0.037 | -0.0668 | No |
| 18 | CASP3 |  |  | 2634 | 0.030 | -0.0919 | No |
| 19 | EHD1 |  |  | 2661 | 0.030 | -0.0906 | No |
| 20 | PDP1 |  |  | 2766 | 0.027 | -0.0985 | No |
| 21 | CALM1 |  |  | 2795 | 0.027 | -0.0979 | No |
| 22 | FN1 |  |  | 2839 | 0.026 | -0.0991 | No |
| 23 | PSMB9 |  |  | 2897 | 0.024 | -0.1022 | No |
| 24 | PIK3CA |  |  | 2955 | 0.023 | -0.1055 | No |
| 25 | MSRB1 |  |  | 2960 | 0.023 | -0.1026 | No |
| 26 | RNF4 |  |  | 3052 | 0.021 | -0.1101 | No |
| 27 | VCPIP1 |  |  | 3210 | 0.018 | -0.1256 | No |
| 28 | GMFB |  |  | 3265 | 0.016 | -0.1295 | No |
| 29 | PPP4C |  |  | 3301 | 0.015 | -0.1313 | No |
| 30 | GPD2 |  |  | 3376 | 0.014 | -0.1378 | No |
| 31 | IRF7 |  |  | 3602 | 0.009 | -0.1623 | No |
| 32 | RABIF |  |  | 3769 | 0.005 | -0.1806 | No |
| 33 | SERPINE1 |  |  | 3811 | 0.004 | -0.1847 | No |
| 34 | CASP7 |  |  | 3926 | 0.002 | -0.1975 | No |
| 35 | PLAUR |  |  | 3956 | 0.001 | -0.2006 | No |
| 36 | GNAI2 |  |  | 4005 | 0.001 | -0.2061 | No |
| 37 | SH2B3 |  |  | 4061 | -0.000 | -0.2123 | No |
| 38 | GNB2 |  |  | 4215 | -0.003 | -0.2294 | No |
| 39 | DYRK2 |  |  | 4485 | -0.008 | -0.2591 | No |
| 40 | S100A13 |  |  | 4664 | -0.012 | -0.2778 | No |
| 41 | CPQ |  |  | 4765 | -0.014 | -0.2874 | No |
| 42 | LAP3 |  |  | 4796 | -0.014 | -0.2888 | No |
| 43 | MMP15 |  |  | 4887 | -0.016 | -0.2967 | No |
| 44 | LGMN |  |  | 4983 | -0.018 | -0.3051 | No |
| 45 | LIPA |  |  | 5191 | -0.022 | -0.3257 | No |
| 46 | AKAP10 |  |  | 5193 | -0.022 | -0.3226 | No |
| 47 | RBSN |  |  | 5301 | -0.024 | -0.3314 | No |
| 48 | PRDM4 |  |  | 5311 | -0.025 | -0.3289 | No |
| 49 | GCA |  |  | 5403 | -0.026 | -0.3356 | No |
| 50 | DOCK10 |  |  | 5405 | -0.026 | -0.3319 | No |
| 51 | PSEN1 |  |  | 5429 | -0.027 | -0.3306 | No |
| 52 | CTSV |  |  | 5544 | -0.029 | -0.3395 | No |
| 53 | USP15 |  |  | 5809 | -0.035 | -0.3649 | No |
| 54 | CA2 |  |  | 5950 | -0.037 | -0.3756 | No |
| 55 | CSRP1 |  |  | 5955 | -0.037 | -0.3707 | No |
| 56 | XPNPEP1 |  |  | 5985 | -0.038 | -0.3686 | No |
| 57 | PLSCR1 |  |  | 6134 | -0.041 | -0.3797 | No |
| 58 | C1R |  |  | 6276 | -0.044 | -0.3896 | No |
| 59 | KIF2A |  |  | 6294 | -0.044 | -0.3851 | No |
| 60 | LTA4H |  |  | 6318 | -0.045 | -0.3813 | No |
| 61 | CPM |  |  | 6334 | -0.045 | -0.3764 | No |
| 62 | L3MBTL4 |  |  | 6537 | -0.051 | -0.3923 | No |
| 63 | CTSO |  |  | 6552 | -0.052 | -0.3865 | No |
| 64 | PREP |  |  | 6667 | -0.055 | -0.3917 | No |
| 65 | PFN1 |  |  | 6817 | -0.059 | -0.4003 | No |
| 66 | RCE1 |  |  | 7001 | -0.065 | -0.4120 | No |
| 67 | CTSD |  |  | 7147 | -0.069 | -0.4187 | Yes |
| 68 | DUSP6 |  |  | 7215 | -0.071 | -0.4161 | Yes |
| 69 | USP14 |  |  | 7294 | -0.074 | -0.4143 | Yes |
| 70 | GNB4 |  |  | 7332 | -0.075 | -0.4077 | Yes |
| 71 | PRCP |  |  | 7495 | -0.081 | -0.4146 | Yes |
| 72 | CASP4 |  |  | 7545 | -0.084 | -0.4082 | Yes |
| 73 | LGALS3 |  |  | 7566 | -0.084 | -0.3983 | Yes |
| 74 | ZEB1 |  |  | 7621 | -0.087 | -0.3920 | Yes |
| 75 | LRP1 |  |  | 7748 | -0.093 | -0.3931 | Yes |
| 76 | GNAI3 |  |  | 7760 | -0.093 | -0.3809 | Yes |
| 77 | TIMP2 |  |  | 7809 | -0.096 | -0.3725 | Yes |
| 78 | CTSB |  |  | 7870 | -0.099 | -0.3650 | Yes |
| 79 | GRB2 |  |  | 7882 | -0.100 | -0.3519 | Yes |
| 80 | USP16 |  |  | 8187 | -0.121 | -0.3694 | Yes |
| 81 | CTSL |  |  | 8250 | -0.126 | -0.3583 | Yes |
| 82 | CLU |  |  | 8387 | -0.145 | -0.3531 | Yes |
| 83 | LAMP2 |  |  | 8425 | -0.149 | -0.3358 | Yes |
| 84 | CD59 |  |  | 8508 | -0.163 | -0.3218 | Yes |
| 85 | COL4A2 |  |  | 8525 | -0.168 | -0.2994 | Yes |
| 86 | CD46 |  |  | 8728 | -0.267 | -0.2842 | Yes |
| 87 | TIMP1 |  |  | 8751 | -0.290 | -0.2449 | Yes |
| 88 | TFPI2 |  |  | 8752 | -0.290 | -0.2031 | Yes |
| 89 | ADAM9 |  |  | 8756 | -0.296 | -0.1608 | Yes |
| 90 | PLAT |  |  | 8772 | -0.327 | -0.1154 | Yes |
| 91 | HSPA5 |  |  | 8785 | -0.369 | -0.0635 | Yes |
| 92 | CTSC |  |  | 8791 | -0.448 | 0.0006 | Yes |
Table: GSEA details [plain text format]

  

Fig 2: HALLMARK\_COMPLEMENT: Random ES distribution      
 Gene set null distribution of ES for **HALLMARK\_COMPLEMENT**
